# Aging-Related Behavioral, Adiposity, and Glucose Impairments and Their Association following Prenatal Alcohol Exposure in the C57BL/6J Mouse

**DOI:** 10.3390/nu14071438

**Published:** 2022-03-30

**Authors:** Susan M. Smith, Eneda Pjetri, Walter B. Friday, Brandon H. Presswood, Dane K. Ricketts, Kathleen R. Walter, Sandra M. Mooney

**Affiliations:** 1 UNC Nutrition Research Institute, University of North Carolina at Chapel Hill, Kannapolis, NC 28081, USA; epjetri@gmail.com (E.P.); walter_friday@unc.edu (W.B.F.); brandon.h.presswood@gmail.com (B.H.P.); danekricketts@gmail.com (D.K.R.); kathleen_walter@unc.edu (K.R.W.); 2 Department of Nutrition, University of North Carolina at Chapel Hill, Kannapolis, NC 28081, USA

**Keywords:** fetal alcohol syndrome, metabolic syndrome, aging, glucose tolerance, developmental origins of health and disease, cognitive performance

## Abstract

People that experience prenatal alcohol exposure (PAE) may have behavioral and metabolic impairments, and it is unclear whether these remain stable or change with age. We assessed behavioral and metabolic endpoints across the lifespan in a mouse model of fetal alcohol spectrum disorder (FASD). Pregnant C57BL/6J mice received alcohol (ALC; 3 g/kg) or maltose-dextrin (control, CON) daily from embryonic day 8.5 to 17.5. Offspring were tested on accelerating rotarod, Y-maze, novel object recognition, and fear conditioning at 6 weeks and 10 and 17 months; females were also tested at 24 months. Body composition, fasting glucose, and glucose clearance were assessed at 18 months. Female but not male ALC mice had greater adiposity than age-matched CON from 7 months onward. At 18 months, male but not female ALC mice had reduced glucose clearance and ALC mice were more likely to have elevated fasting glucose. In the rotarod training session, ALC females performed worse than CON. In the Y-maze, significant exposure-age interactions affected ALC performance in both sexes versus age-match CON. For fear conditioning, all animals acquired the task and froze more at older ages. In both the context and cued tasks, there were exposure-age interactions and ALC animals frozen less than CON at 10 months. Correlation analysis revealed that fasting glucose and glucose clearance correlated with % of body fat in ALC but not in CON mice. Additionally, glucose intolerance and % body fat negatively correlated with performance in the rotarod, context learning, and novel object recognition tasks in ALC but not CON mice. All mice exhibit worsening of behavioral performance as they age, and PAE did not further exacerbate this. ALC but not CON mice displayed adiposity and glucose intolerance that correlate with their cognitive impairments, suggesting that these may be mechanistically related in PAE. Findings emphasize that FASD should be considered a whole-body disorder.

## 1. Introduction

Prenatal alcohol exposure (PAE) causes a characteristic constellation of behavioral, cognitive, growth, and morphological deficits collectively known as fetal alcohol spectrum disorders (FASDs) [1]. In the U.S., 13.5% of pregnant women consume alcohol and 5.2% self-report binge drinking [2], and between 1–5% of first graders meet the criteria for FASD, based on an epidemiological survey of four U.S. communities [3]. Thus, affected individuals represent a significant segment of the population. Although these deficits persist across the lifespan, studies have typically focused on children and adolescents, and thus our understanding of their impact in later life is limited [4,5,6,7].

All organ systems, including the central nervous system, experience aging, or biological change over the lifespan, and these changes follow a predictable, progressive timeline [8]. This progression is modified by interactions with environmental factors, including alcohol [9]. Early onset of this decline reduces the ‘healthspan’, or time free from chronic disease [10]. The timing of this onset varies individually and is influenced by both genes and environment.

The limited ‘healthspan’ information we do have for adults with PAE suggests they may be at greater risk, and with earlier onset, for chronic diseases that typically emerge later in life. A recent self-study of 541 adults (mean age 27.5 years) with known PAE revealed extraordinarily high levels of autoimmune disorders, cardiovascular problems, endocrine disorders including diabetes and thyroid disease, mental health disorders, and early cognitive decline [11]. In a chart review from an academic healthcare system, males diagnosed with FASD (mean age 29 years) had higher rates of type-2 diabetes despite normal body-mass indices (BMIs) whereas their female counterparts had elevated BMIs, and both had elevated plasma triglycerides and reduced HDL [12]. Castells et al. [13] described elevated plasma insulin in an oral glucose tolerance test in children with fetal alcohol syndrome, suggesting they had greater insulin resistance. In contrast, a cohort of mostly African Americans with FASD (mean age 36 years) had lower mean BMIs and no difference in their diabetic indicators (fasting glucose, HOMA-IR); although, they had a steeper slope when BMI was plotted against fasting insulin [14]. Although children diagnosed with FASD are more typically lean or even underweight, likely because small-for-age is part of the diagnostic criteria [1], elevated adiposity is more frequently observed in females, but not males, starting around the time of adolescence [12,15,16,17,18]. There may also be lasting elevations in inflammatory indices, which are independent risk factors for diabetes and obesity [19,20,21,22]. These metabolic findings are supported by preclinical investigations, which consistently report that PAE worsens the hallmarks of metabolic syndrome including adiposity, diabetes, hypertension, and cardiovascular disease [18,23]. As few clinical studies report cognitive performance across the lifespan, it is unknown if the cognitive deficits caused by PAE remain stable, improve, or worsen with advancing age. Preclinical studies take their cue from this work and seldom investigate animals beyond adolescence or young adulthood.

To address this knowledge gap, we perform a progressive assessment of learning and behavior in a mouse model of moderate PAE [24]. These animals may model alcohol-related neurodevelopmental disorder (ARND) and at adolescence they show deficits in associative memory and hyper-responsivity with no apparent effects on growth or morphology. Here, we assess behaviors in these same animals during adulthood and into old age to determine whether their behavioral deficits remain static, improve, or worsen with age. We also assess their body composition and glucose handling in later adulthood and examine the potential association of these with the cognitive outcomes.

## 2. Materials and Methods

### 2.1. Mice and Husbandry

Mice were generated as described previously [24,25]. Five-week-old C57BL/6J mice (Jackson Laboratories, Bar Harbor, ME, USA) were housed in a humidity- and temperature-controlled facility with a 12-hour light–dark cycle (on 7 a.m., off 7 p.m.). Animals consumed a purified, nutritionally adequate diet (AIN-93G; Teklad Envigo, Madison, WI, USA; [26]) throughout the study period. At an age of 8 weeks, females were mated overnight, and the morning of plug detection was defined as embryonic day (E) 0.5 (E0.5). Daily from E8.5 through E17.5, pregnant females received a single oral gavage of 3.0 g/kg alcohol (ALC), or maltodextrin (control, CON) (Teklad Envigo, Madison, WI, USA) at 80% of alcohol calories; higher maltodextrin doses were hyperosmotic and injurious to the mouse. This represents a difference of 0.1 kcal/d and 1.01% of total daily calories. Pregnant dams were weighed daily and typically gave birth on E18.5. This study used 7 CON dams and 10 ALC dams to generate 16 CON males, 14 CON females, 29 ALC males, and 15 ALC females. Blood alcohol concentrations in this model were 211 ± 14 mg/dL 30 min post-gavage [24].

Offspring were weighed weekly until weaning at postnatal day (P)22, monthly through age 11 months, and bi-weekly or weekly thereafter. Mice were assessed weekly for signs of illness; malocclusions and nails were trimmed as needed. Mortality and cause of death (when it could be determined through necropsy) were recorded; surviving male and female mice were euthanized at an age of 21 months (~637 days) and 24 months (~726 days), respectively. Mice that lost >10% body weight in a one-week period were humanely euthanized. All protocols were approved by the Institutional Animal Care and Use Committee of the David H. Murdock Research Institute.

### 2.2. Behavioral Assessments

At ages 6 weeks, 10 months, 17 months, and 24 months (females only), mice underwent behavioral testing. At an age of 6 weeks, we sampled one male and one female per litter. At older ages, we tested all remaining offspring in the litter; Supplemental Table S1 presents the number of animals tested at each age. The testing order was accelerating rotarod, the Y-maze test of spontaneous alternation, the novel object recognition test, and contextual- and cued auditory fear conditioning. Data from 6-week-old animals have previously been published [24] but are included here to allow assessment across multiple ages.

Rotarod. Mice were placed on a cylinder that slowly accelerated from 3 revolutions per min (rpm) to 30 rpm in 5 min. Mice received a training trial (trial 1, T1) and, after a 45 s break, the test trial (T2). Latency (s) to fall was recorded for both trials.

Spontaneous Alternation in Y-Maze. A Y-shaped apparatus was used, having three symmetrical arms at a 120-degree angle (30 cm long, 8 cm wide, and 15 cm high). Mice were placed in the center and left to freely explore the maze for 8 min. The number of arm entries was quantified using Ethovision XT software (Noldus Information Technology, Leesburg, VA, USA). Dependent measures were number of visits (arm entries), number of alternations (entering all three arms without reentering one), and the percent of alternations ((number of alternations/number of visits − 2) × 100).

Activity. Mice were placed in an open, rectangular arena (51 cm × 28 cm; height 27.5 cm) for 2 min. Movement distance was quantified using Ethovision XT software.

Novel Object Recognition. Mice were reintroduced to the arena above now containing two plastic eggs (4 cm × 5.5 cm) or two 20 mL glass vials (6 cm × 2.5 cm) weighted with sand. Objects were placed equally distant from each other and from the walls of the arena. Mice were allowed to explore the objects for 3 min. Twenty-four hours later, the mouse was placed in the arena with one familiar object from day one and one novel object and left to explore for three min. The dependent measure was % time exploring the novel object (time exploring novel/(time exploring novel + time exploring familiar) × 100). Animals that spent less than 2 s exploring objects were excluded from analysis. This test was performed at 10, 17, and 24 months but not at 6 weeks.

Fear Conditioning. Acquisition. After 2 min in a conditioning chamber, a tone (conditioned stimulus (CS); 2.8 kHz, 85 db) was played for 30 s. For the last 2 s of the CS a mild foot shock (unconditioned stimulus (US); 0.75 mA) was delivered. The CSUS pairing was repeated four times with an intertrial interval of 80 s, then the mouse remained in the chamber for another 80 s. Contextual fear testing. Twenty-four hours later, the mouse was placed in the same conditioning chamber for 5 min. Cued fear testing. Twenty-four hours after contextual fear testing, the mouse was placed in an altered chamber (the patterns on the walls and the floor were changed and a vanilla odor was introduced). After 2 min, the CS was played for 3 min. For all three testing phases, freezing behavior—defined as total lack of movement except for respiration—was recorded for each minute.

### 2.3. Metabolic Assessment

Body Composition was quantified at 18 months of age, at the conclusion of the 17-month behavioral testing, using non-invasive NMR (EchoMRI 4-in-1-1100 Analyzer, Houston, TX, USA), which provides fat mass, lean mass, and total water. Percentages of fat mass, lean mass, and water mass were calculated using body weight taken on the same day.

Glucose tolerance was quantified in male and female mice following the body composition analysis using an intraperitoneal glucose tolerance test (IPGTT; [27]). Mice were fasted for 5 h (8 a.m.–1 p.m.) in a new cage with continued access to water. Glucose levels were measured in blood obtained from a tail clip using a handheld glucometer (One-Touch Ultra2; 5 µL sample) prior to and 15, 30, 60, 90, and 120 min after an i.p. injection of glucose (1.0 g/kg body weight as a 10% solution in PBS). Fasting glucose and glucose clearance were determined, calculating the latter as area-under-the-curve (AUC) using the trapezoidal rule.

### 2.4. Statistical Analysis

Dam and offspring body weights were analyzed by repeated measures analysis of variance (ANOVA) with follow-up 2-way ANOVA within sex (exposure and age as factors). Body composition data were analyzed by 2-way ANOVA (exposure and sex as factors), where sex was significant, follow-up 1-way ANOVAs were run. IPGTT data were analyzed by 3-way ANOVA. Fisher’s exact test was used to determine between group differences in the proportion of (pre)diabetic animals. The Log Rank test was used to analyze survival data.

For behavior, the number of animals of the same sex used from each litter was variable (*n* = 0–4) and not all animals were tested at all ages, therefore, we used a linear mixed effects model (LMEM) with fixed variables of exposure (ALC, CON), sex, age, and a random variable of litter. Data for acquisition of fear contains two repeats (time and age), thus, we used LMEM to look at each minute within each age and then to look at each stage across all ages. Post hoc analyses used Bonferroni t-test. Alpha was set at <0.05, trends are reported if *p* < 0.1. Linear regression was used to test for associations among metabolic outcomes and between metabolic and behavior outcomes. Data analysis was performed using SigmaPlot (ver. 12, Systat Software Inc., San Jose, CA, USA) or SPSS (IBM SPSS Statistics software, ver. 26, Armonk, NY, USA).

## 3. Results

### 3.1. Growth and Metabolic Outcomes

#### 3.1.1. Body Weight

**Figure 1:**
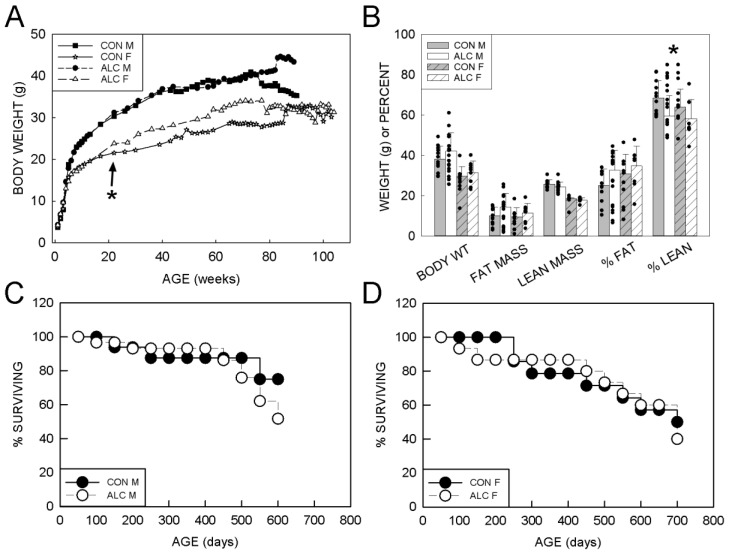
Body weights, body composition, and survival of ALC and CON mice. (**A**) Body weights of males (closed symbols) and females (open symbols). ALC females are heavier (*p* < 0.001) than CON from 200 days onward (arrow). (**B**) Body composition for 18-month-old CON and ALC mice; lean mass and fat mass are presented as both absolute weights (g) and as a percentage of body weight. (**C**) Percent survival of male ALC and CON mice. (**D**) Percent survival of female ALC and CON mice. N = 10–12 F and 16–22 M per group. * Indicates ALC differs from CON at *p* ≤ 0.05. ALC—alcohol-exposed. CON—control. F—female. M—male.

As reported previously for this cohort [24], absolute body weight of the maternal CON and ALC mice did not differ until late pregnancy, when ALC dams weighed less at E16.5 and E17.5 (Supplemental Table S2). ALC dams gained less weight across the pregnancy (E0.5–E17.5) and during the gavage period (E8.5–E17.5) than CON animals. Both male and female ALC pups weighed more than CON at P9, P15, and P22. Offspring body weights showed an exposure × sex × age interaction (F_66, 2298_ = 1.415, *p* = 0.016), a sex × age interaction (F_56, 2298_ = 3.433, *p* < 0.001), and main effects of effect of age (F_72, 2298_ = 89.958, *p* < 0.001) and sex (F_1, 2309_ = 1115.927, *p* < 0.001; M > F). Litter accounted for 25% of the variance in weight data. Body weights for males and females diverged after ~6 weeks of age (Figure 1A). Follow-up analyses used 2-way ANOVA (exposure, age) separately for males and females: In the male offspring there was a significant effect of exposure (F_1, 971_ = 7.212, *p* = 0.007) and age (F_28, 971_ = 93.506, *p* < 0.001), but no interaction; body weight increased with age for both groups, and ALC males were heavier than CON. The effect of exposure in these males was driven by changes that occurred after 75 weeks of age, when ALC males with lower body weights and CON males with higher body weights began to die off. Prior to 75 weeks, ALC and CON males had similar weights. Female data also showed a significant main effect of age (F_42, 757_ = 40.597, *p* < 0.001) and exposure (F_1, 757_ = 17.84, *p* < 0.001). Again, body weight increased with age for both groups, and ALC females were heavier than CON.

#### 3.1.2. Body Composition

A 2-way ANOVA analysis of the body composition analysis performed between 77 and 80 weeks of age (18 months) did not identify a significant difference between groups in fat mass (g) or % body fat; however, lean mass (g) was higher in males than females (F_1, 45_ = 129.076, *p* < 0.001) and % lean mass was significantly higher in CON than ALC (F_1, 45_ = 5.154, *p* = 0.028) (Figure 1B). One-way analyses within sex found that absolute lean mass was significantly higher in CON males than ALC males (*p* = 0.038), and that % lean mass showed a trend to be higher in CON females than ALC females (*p* = 0.064).

#### 3.1.3. Glucose Tolerance

**Figure 2:**
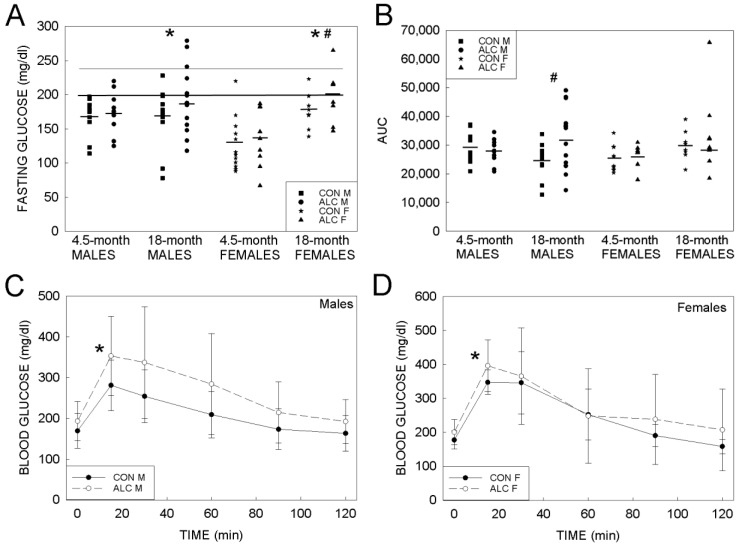
Metabolic responses in 18-month-old ALC and CON mice. (**A**) Fasting blood glucose at an age of 4.5 months and 18 months. At an age of 4.5 months, fasting glucose does not differ between CON and ALC males and females. At an age of 18 months, ALC females have higher fasting glucose than 4.5-month-old ALC females. Mean fasting glucose was greater in ALC than CON offspring. Lower line indicates fasting blood glucose ≥ 200 mg/dL (prediabetes) and upper line ≥ 240 mg/dL (diabetes). Symbols represent values for individual animals; bars indicate the mean response. (**B**) Glucose clearance in the intraperitoneal glucose tolerance test, presented as area-under-the-curve (AUC). Symbols represent values for individual animals; bars indicate the mean response. At 4.5 months, AUC does not differ by exposure or sex. At 18 months, male ALC mice had higher AUCs than 4.5-month-old ALC mice and CON males at both ages, suggesting reduced glucose clearance. The four 18-mo ALC mice having AUC > 40,000 also have fasting glucose ≥ 240 mg/dL in (**A**). (**C**,**D**) Blood glucose levels following intraperitoneal injection of glucose into fasting males (**C**) and females (**D**). Mean blood glucose levels were higher in ALC mice than CON. Values are mean ± S.D. Values for 4.5-month-old mice are taken from Amos-Kroohs et al. (2018) and are included for comparative purposes. *, *p* ≤ 0.05 for age- and sex-matched ALC versus CON. #, *p* ≤ 0.05 for sex-matched 18-mo versus 4.5-mo ALC mice.

The elevated weight and lower lean mass of the ALC animals suggested these animals might have worsened glucose tolerance, which has been reported in other PAE models [28]. In our prior work with mice that were generated using this model [27], mean fasting glucose values at young adulthood (4.5-months of age) did not differ between ALC and CON male and female offspring. In the present study, 3-way ANOVA revealed significant age × sex interaction (F_1, 76_ = 6.764, *p* = 0.011) and a main effect of age (F_1, 76_ = 15.523, *p* < 0.001) wherein 18-month-old ALC females had higher fasting glucose than 4.5-month-old females (Figure 2A). For the males, fasting glucose values for the ALC and CON did not differ at 18 months, and also did not differ from values reported previously for their 4.5-month-old counterparts [27]. The ANOVA also identified trends for exposure (F_1, 76_ = 2.790, *p* = 0.099) and sex (F_1, 76_ = 3.127, *p* = 0.081), such that mean fasting glucose was greater for ALC than CON offspring and was greater for males than for females.

Unlike for humans, there are not uniform criteria for diabetes in mouse, and suggested cutoffs for blood glucose after a 6 h fast range from ≥126 mg/dL to ≥250 mg/dL [29,30]. Using a conservative set of cutoffs, at an age of 18 months 45.5% of ALC mice (10/22) and 15% of CON mice (3/20) met the criteria for prediabetes (fasting blood glucose ≥ 200 mg/dL) or diabetes (≥240 mg/dL), a difference that was significant (*p* = 0.009). The population was too small to distinguish a sex effect.

With respect to glucose clearance following intraperitoneal glucose administration, as reflected in the area-under-the-curve (AUC), 3-way ANOVA identified trends for an exposure × age interaction (F_1, 76_ = 3.061, *p* = 0.084) and for a main effect of age (F_1, 76_ = 3.121, *p* = 0.081) wherein 18-month-old ALC animals had poorer clearance than did CON animals or 4.5-mo ALC animals (Figure 2B). Two-way ANOVA within sex found that in males there was a significant exposure × age interaction (F_1, 42_ = 4.671, *p* = 0.036), such that 18-month-old ALC animals had poorer glucose clearance than did 4.5-month-old ALC males. In contrast, age did not alter glucose clearance in the CON males. In females, the 2-way ANOVA only identified a main effect of age (F_1, 34_ = 5.046, *p* = 0.031), wherein 18-mo animals cleared the glucose challenge slower than did the 4.5-month-old females, and PAE did not further affect this. The time-course analysis affirmed these findings, and a 2-way repeated measures ANOVA identified a significant main effect of exposure (F_1, 205_ = 4.232, *p* = 0.046) and time (F_5, 205_ = 79.486, *p* < 0.001), such that blood glucose levels were higher in ALC mice than CON (Figure 2C,D). The 4 18-month-old ALC mice having AUCs > 40,000 also had fasting glucose ≥ 240 mg/dL (Figure 2A).

#### 3.1.4. Survival

Log Rank analysis in Kaplan–Meier plots reveal that survival of CON and ALC animals did not differ (Figure 1C). However, males in both groups experienced a 12% loss of survival between 500 and 550 days, and ALC males had another 10% loss between 550 and 600 days leaving only 50% of the original ALC cohort by this age. Thus, the male cohort was terminated at an age of 637 days (21 months), and the female cohort continued to 726 days (24 months).

### 3.2. Behavioral Assessment

The LMEM outcomes showed that for the behavior metrics, litter only explained between 0% and 11% of the variability and was typically ≤ 5% (Supplemental Table S3), and thus litter was not considered to be a major contributor to these outcomes.

#### 3.2.1. Rotarod

**Figure 3:**
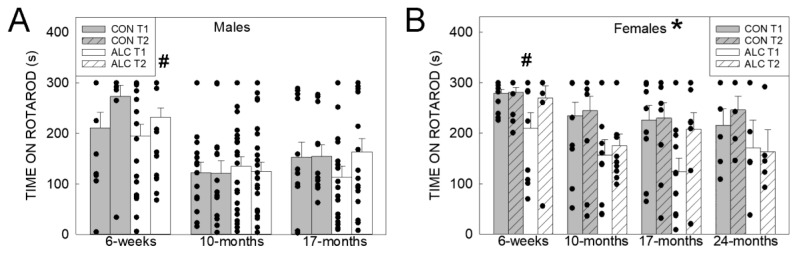
ALC and CON performance on accelerating rotarod. (**A**) Six-week-old males performed better than at older ages. No other differences were found. (**B**) ALC females perform worse than age-matched CON females, and 6-week-old females performed better than at older ages. Values are mean ± SEM, dots represent values for individual animals. The number of animals tested in each group is presented in Supplemental Table S1. T1, training trial; T2, test trial. * Indicates ALC differs from CON at *p* ≤ 0.05. # Indicates difference between 6 weeks vs. older ages, *p* < 0.01.

For Trial 1 on the rotarod, LMEM analysis identified a significant exposure × sex interaction (F_1, 166_ = 5.396, *p* = 0.021) as well as main effects of exposure (F_1, 6_ = 9.207, *p* = 0.021), sex (F_1, 166_ = 12.066, *p* = 0.001), age (F_3, 165_ = 7.045, *p* < 0.001) (Figure 3A,B). ALC females performed worse than CON females (*p* = 0.002), whereas there were no differences between ALC and CON males (*p* = 0.531). CON females outperformed CON males (*p* < 0.001), but there were no differences between sexes in the ALC animals (*p* = 0.353). Six-week-old animals outperformed older animals. For Trial 2, LMEM analysis only identified significant main effects of sex (F_1, 175_ = 15.998, *p* < 0.001) and age (F_3, 167_ = 15.043, *p* < 0.001). Females outperformed males, and 6-week-old animals outperformed older animals.

#### 3.2.2. Y-Maze

**Figure 4:**
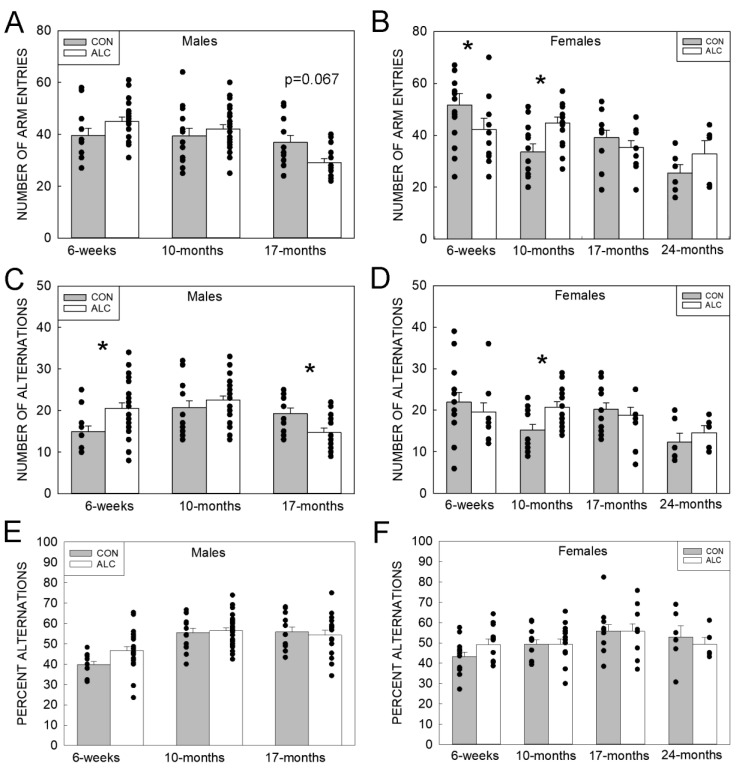
ALC and CON performance on the Y-maze task. (**A**,**B**) Number of arm entries does not differ in males (**A**), and differs in ALC and CON females at 6 weeks and 10 months. (**C**,**D**) The number of arm alternations differs in ALC and CON males at 6 weeks and 17-mo (**C**), and differs in ALC and CON females at 10 months (**D**). (**E**,**F**) The percentage of alternations did not differ by exposure in males (**E**) and females (**F**) at all ages tested. Values are mean ± SEM, dots represent values for individual animals. The number of animals tested in each group is presented in Supplemental Table S1. * Indicates ALC differs from CON at *p* ≤ 0.05.

For the number of arm entries there was a significant exposure × sex × age interaction (F_5, 165 =_ 2.804, *p* = 0.019), a significant exposure × age interaction (F_3, 163_ = 4.510, *p* = 0.005), a significant main effect of age (F_3, 163_ = 13.055, *p* < 0.001), and a trend for main effect of sex (F_1, 170_ = 3.419, *p* = 0.066). Males showed a trend (*p* = 0.067) for CON having a greater number of arm entries than ALC at 17 months, with no differences at other ages (Figure 4A). In females there was a significant difference between CON and ALC at 6 weeks (CON > ALC) and 10 months (ALC > CON) but not at the older ages (Figure 4B). For the number of alternations there were significant exposure × sex × age (F_3, 169_ = 2.803, *p* = 0.041), sex × age (F_2, 164_ = 5.655, *p* = 0.004), and exposure × age (F_3, 164_ = 3.033, *p* = 0.031) interactions, and a main effect of age (F_3, 165_ = 4.030, *p* = 0.008). For the males, ALC had more alternations than CON at 6 weeks, whereas CON had more than ALC at 17 months (Figure 4C). For the females, the number of alternations was greater in CON than ALC at 10 months (Figure 4D). For the percent alternations, there was a sex × age interaction (F_2, 176_ = 3.829, *p* = 0.024) and a significant main effect of age (F_3, 176_ = 12.345, *p* < 0.001); percent of alternations by males was greater than females at 10 months (*p* = 0.005) but not at 6 weeks or 17 months (Figure 4E,F).

#### 3.2.3. Novel Object Recognition

Activity during acclimation to the test arena was only affected by age (F_2, 116_ = 15.589, *p* < 0.001), with 10-month-old animals more active than at 17 months. There were no significant effects of exposure, sex, or age on percent time exploring the novel object after a 24-hour waiting period (Supplemental Figure S1).

#### 3.2.4. Fear Conditioning

During the Acquisition phase (Day 1), the within-age comparisons always identified time as significant (*p* < 0.001) as freezing increased across repeated experiences of the CSUS pairing, suggesting the animals learned that the tone predicted the shock. The main effect of sex was significant at an age of 6 weeks (F_1,261_ = 18.944, *p* < 0.001) and 17 months (F_1, 244_ = 21.356, *p* < 0.001), and was a trend at 10-mo (F_1, 289 =_ 3.095, *p* = 0.080), with females freezing more than males (Supplemental Figure S2A–D). Comparison of behavior within stage (min) across all ages identified age differences (main effects and/or interactions) for all stages. During the acclimation period prior to tone presentation (background), there was a main effect of age (F_3, 166_ = 23.55, *p* < 0.001); 17- and 24-month-old mice froze more than 6-week-old and 10-month-old animals. A similar age effect emerged in the CSUS1 (F_3, 175_ = 30.288, *p* < 0.001) such that freezing was the least at 6 weeks, was greater at 10 months and 17 months, and was greatest at 24 months. For CSUS2, there were main effects of exposure (F_1, 8_ = 7.416, *p* = 0.025) wherein ALC froze less than CON, as well as of age (F_3, 168_ = 38.574, *p* < 0.001) wherein freezing was again lowest at 6 weeks, and a trend for sex (F_1, 175_ = 3.494, *p* = 0.063; F > M). For CSUS3. there were main effects of age (F_3, 170_ = 19.599, *p* < 0.001; less freezing at 6-wk) and sex (F_1, 176_ = 15.029, *p* < 0.001; F > M). For CSUS4, there was a sex × age interaction (F_2, 166_ = 3.716; *p* = 0.026); females froze more than males at 6 weeks and 17 months, but not at 10 months, and a main effect of sex (F_1, 174_ = 18.108, *p* < 0.001; F > M).

**Figure 5:**
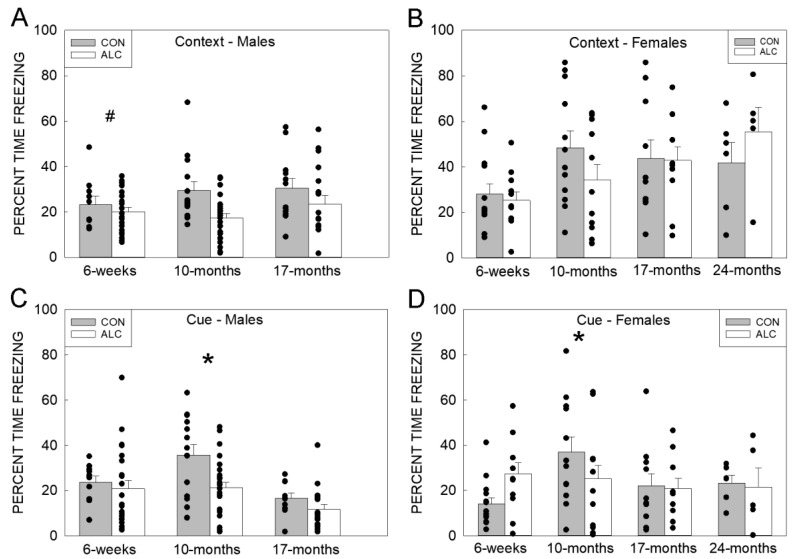
ALC and CON performance in the contextual and cued fear conditioning task. (**A**,**B**) Percent time freezing does not differ in ALC and CON males (**A**) or females (**B**) in the contextual learning task. There is less freezing in males at 6 weeks versus older ages. (**C**,**D**) Percent time freezing is reduced in ALC males at 10 months (**C**) and in ALC females at 10 months (**D**) compared with age-matched CON in the cued learning task. Values are mean ± SEM, dots represent values for individual animals. The number of animals tested in each group is presented in Supplemental Table S1. * Indicates ALC differs from CON at *p* ≤ 0.05. # Indicates that 6 weeks differs from older ages at *p* < 0.05.

In the test of Context learning (Day 2), total context (average % time freezing over the 5 min) showed significant effects of age (F_3, 161_ = 5.406, *p* = 0.001) and sex (F_1, 163_ =24.845, *p* < 0.001); freezing was higher in adults than adolescent animals and higher in females than in males (Figure 5A,B; Supplemental Figure S2E–H). There were also trends for interactions for exposure × age (F_3, 161_ = 2.308, *p* = 0.079) and sex × age (F_2, 160_ = 2.535, *p* = 0.082). Repeated measures ANOVA of the % time freezing minute-by-minute within each age identified sex differences at all three ages such that females froze more than males (smallest F was at 6 weeks: F_1, 265_ = 7.821, *p* = 0.006). No other effects were seen in adolescent animals. At an age of 10 months, there were effects of time (F_4, 293_ = 6.629; *p* < 0.001), with the lowest freezing during the first minute (highest *p* = 0.025), and exposure (F_1, 10_ = 6.764, *p* = 0.026), with ALC animals freezing less than CON animals. There was also time (F_4, 224_ = 3.605, *p* = 0.007) but no exposure effects at an age of 17 months; freezing during min 1 was significantly lower than min 2 (*p* = 0.022) and 3 (*p* = 0.009) and tended to be lower at min 5 (*p* = 0.083). At 24 months, there was a trend for an effect of time (F_4, 38_ = 2.381, *p* = 0.068) but no exposure effect.

In the Cue test, total cue (average % time freezing during the 3 min CS), showed an age × exposure interaction (F_3, 159_ = 3.080, *p* = 0.029) with ALC freezing less than CON at an age of 10 months, and an effect of age (F_3, 159_ = 6.541, *p* < 0.001) (Figure 5C,D; Supplemental Figure S2I–L). Repeated measures ANOVA within each age found for 6-week-old animals an exposure × sex interaction (F_1, 260_ = 6.242, *p* = 0.013) in which ALC females, but not males, showed greater freezing than sex-matched CON, as well as effects of sex (F_1, 260_ = 4.134, *p* = 0.043; M > F) and time (F_4, 254_ = 26.850, *p* < 0.001); freezing during min 3 was higher than at all other times. Animals at an age of 10 months showed a significant exposure × time interaction (F_4, 293_ = 2.647, *p* = 0.034), such that ALC had less freezing than CON at min 3, 4, and 5 (highest *p* = 0.030). There were also effects of time (F_4, 293_ = 60.377, *p* < 0.001), wherein freezing during min 3 was different to min 1, 2, and 5 but not min 4, sex (F_1, 305_ = 4.722, *p* = 0.031), wherein females froze more than males, and exposure (F_1, 10_ = 5.194, *p* = 0.046) wherein ALC froze less than CON. The older adults (17 months) showed effects of sex (F_1, 220_ = 18.511, *p* < 0.001; F > M) and time (F_4, 207_ = 27.788, *p* < 0.001), but no effect of exposure. Freezing during min 3 was different to min 1, 2, and 5 but not min 4. In aged females (24 months), only time showed an effect (F_4, 37_ = 7.715, *p* < 0.001); freezing during min 1, 2, and 3 was lower than min 4 and 5, suggesting a delayed response to the cue.

### 3.3. Association Analyses

**Figure 6:**
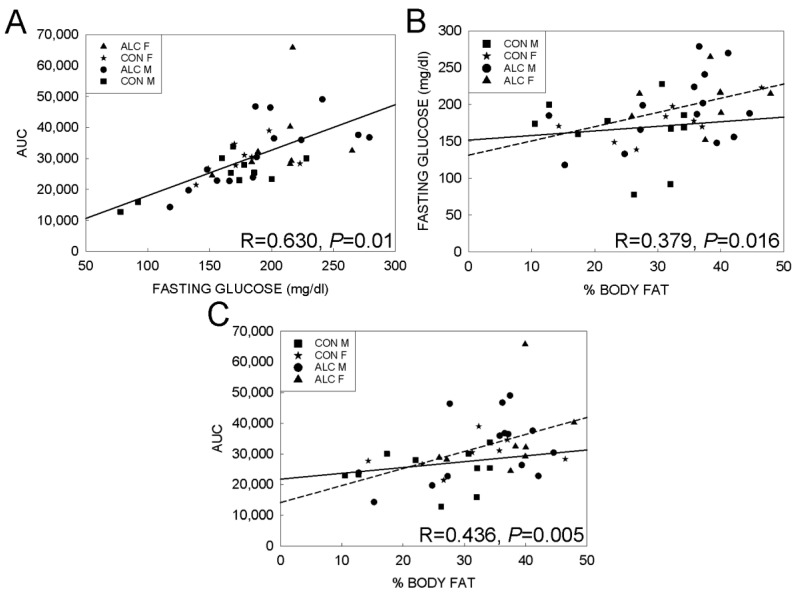
Association analysis between % body fat, fasting glucose, and glucose tolerance in 18-mo ALC and CON mice. (**A**) Linear regression shows a positive correlation between fasting glucose and AUC in all mice. (**B**) The correlation between % body fat and fasting glucose is positive for ALC mice but is not significant for CON mice. (**C**) The correlation between % body fat and AUC is positive for ALC mice but not for CON mice. For (**B**,**C**), lines represent the correlations for CON (solid) and ALC (dashed) mice; R values are for ALC mice only, as CON R values were not significant (see text for details). Symbols represent individual animals.

We first examined the associations among the metabolic measures. Consistent with their known mechanistic relationships, AUC in the IPGTT showed a strong, positive correlation with fasting glucose in all animals (R = 0.630, *p* < 0.001) and this was not further modified by exposure (Figure 6A). Fasting glucose (R = 0.379, *p* = 0.016; Figure 6B) and AUC (R = 0.436, *p* = 0.005; Figure 6C) were also correlated with % body fat and their association showed greater variance. Reanalysis revealed that these associations were dependent on exposure, such that fasting glucose (R = 0.407, *p* = 0.06) and AUC (R = 0.434, *p* = 0.044) correlated with % body fat in the ALC mice, but not in CON mice (*p* = 0.537 and *p* = 0.256, respectively), suggesting that the relationship between fat mass and glucose clearance was different in the PAE animals compared with CON mice. Skeletal muscle is a primary site for glucose clearance, and reanalysis using % lean mass as the independent variable revealed that this association was weaker as compared with % fat mass for the ALC mice (fasting glucose R = 0.382, *p* = 0.08; AUC R = 0.418, *p* = 0.053) and showed no association for CON (fasting glucose R = 0.147, *p* = 0.560; AUC R = 0.225, *p* = 0.369).

**Figure 7:**
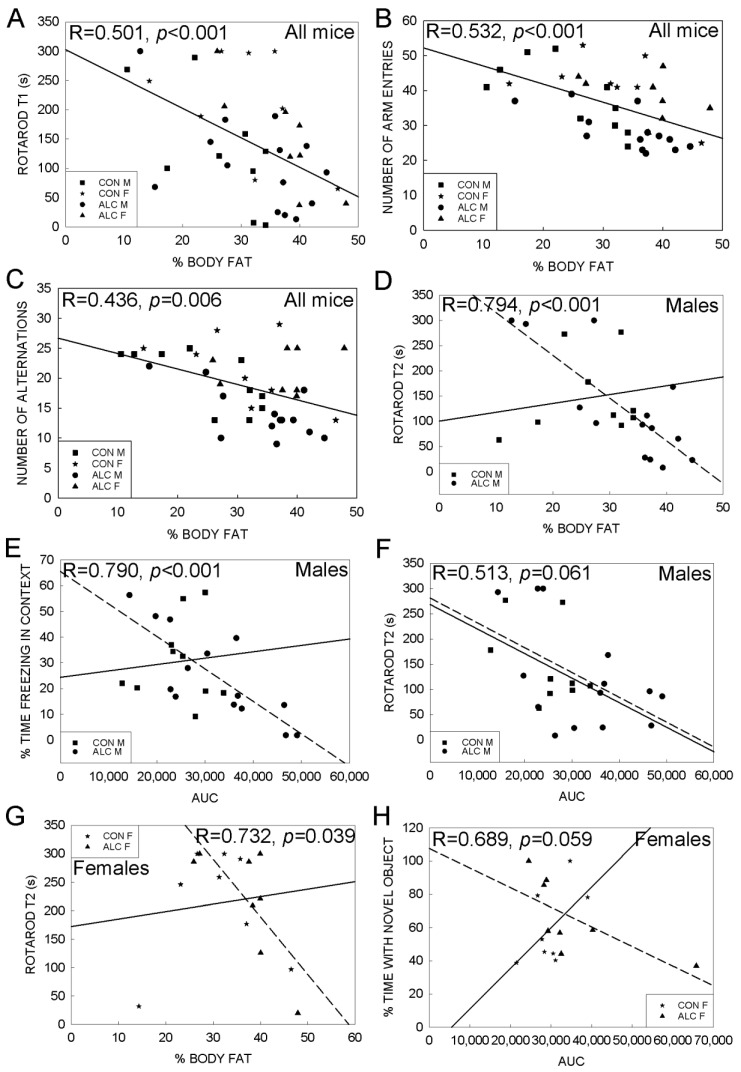
Association analysis between metabolic and behavioral outcomes in 18-month-old ALC and CON mice. (**A**) Regression analysis shows a negative correlation between % fat mass and rotarod training (T1) performance at 17 months in all mice. Correlation between % fat mass and number of arm entries (**B**) and the number of alternations (**C**) in the Y-maze test is also negative for all mice. (**D**) Correlation between % fat mass and rotarod performance at trial 2 (T2) is negative for ALC males but not significant for CON males. (**E**) A negative correlation between AUC and % time freezing in the contextual fear conditioning task is seen in ALC male mice, but not CON males. (**F**) In the rotarod trial (T2), the correlation with AUC is negative for ALC males but not CON males. (**G**) In females, there is a negative correlation between % fat mass and rotarod performance at trial 2 (T2) in ALC mice but not CON. (**H**) There is a trend for a negative correlation between AUC and % time spent with the novel object for ALC female mice but not in CON females. In all panels, symbols indicate values for individual animals. Regression lines for CON are solid and are dashed for ALC. Only regression values that were significant are presented; in (**A**–**C**) these are for all mice and in (**D**–**H**) only ALC values are significant.

Type-2 diabetes is an independent risk factors for cognitive decline and lower performance on tasks of learning and memory [31,32]. We thus extended the analysis to evaluate potential interactions between these metabolic measures and the behavioral outcomes when both were performed in the same animals. This revealed associations between select behavioral outcomes and % fat mass and AUC, but not with fasting glucose or % lean mass. For the population overall, % fat mass inversely correlated with T1 performance on the rotarod (R = 0.501, *p* < 0.001; Figure 7A), and the number of visits (R = 0.532, *p* < 0.001; Figure 7B) and alternations (R = 0.436, *p* = 0.006; Figure 7C) in the Y-maze, and these were not further affected by exposure or sex. No additional correlations were identified in the male or female CON mice. In contrast, % fat and AUC inversely correlated with learning only in the ALC mice. In the ALC males, % fat negatively correlated with T2 performance in the rotarod (R = 0.794, *p* < 0.001; Figure 7D), and AUC negatively correlated with freezing in the context fear conditioning task (R = 0.790, *p* < 0.001; Figure 7E) and trended to negatively correlate with T2 performance (R = 0.513, *p* = 0.061; Figure 7F). In the ALC females, % fat mass again negatively correlated with T2 performance (R = 0.732, *p* = 0.039; Figure 7G) and AUC tended to negatively correlate with time spent in novel object exploration (R = 0.689, *p* = 0.059; Figure 7H).

## 4. Discussion

This preclinical, prospective longitudinal study addresses a significant knowledge gap in the FASD literature: do the cognitive and pathological sequelae of PAE worsen, improve, or remain static with age? With respect to behaviors, although these generally deteriorated with advancing age, few interactions emerged between exposure and age. This suggested that the PAE animals did not experience a greater cognitive decline as they aged, and this lack of age-related change was true for all behaviors, regardless of whether the behavior was affected by PAE when the animals were first assessed at adolescence (age of 6 weeks). This behavioral stability across their lifespan contrasts with the metabolic measures for these same animals, which exhibited significant age-exposure interactions such that the ALC animals were more likely to exhibit metabolic dysfunction by the age of 18 months. The ALC females were heavier from 7 months onward despite their reduced lean mass and this likely represented increased adiposity. The ALC males at 18 months had worsened glucose clearance compared against 4.5-month-old ALC males and CON at both ages. These data suggest that ALC animals were at greater risk for type-2 diabetes as they aged.

Novel insights emerged from this study’s unique assessment of both behavior and glucose handling in the same animals. This revealed that declines in learning correlated with an individual’s adiposity and ability to clear a glucose challenge. We speculate that these factors—adiposity and glucose intolerance—contribute to some of the variance in behavioral responses, and perhaps other outcomes, in those with FASD. It emphasizes that FASD should be considered as a whole-body disorder, and it further suggests that the magnitude of cognitive impairment in these animals may be mechanistically related to their metabolic deterioration. It also introduces the speculation that, as reported elsewhere for type-2 diabetes, interventions that improve glucose metabolism, such as medications and exercise, could forestall additional cognitive decline.

Longevity of ALC offspring appeared to be unaffected by PAE in this model, although the study was underpowered to test this rigorously. When possible, a veterinarian performed gross necropsies, and although cause-of-death could not always be determined, deaths due to fight injuries occurred almost uniquely in the ALC males (6 vs. 1). We also identified three cases of cancer (blood, testicular, and lipoma), two cardiac enlargements, and three instances of gastrointestinal hemorrhage, and all were limited to ALC animals. It cannot be determined if these represent a true difference in disease risk, and a larger study accompanied by diagnostic pathology will be necessary to make this determination.

Consistent with prior reports in other animal models of PAE, we find that glucose handling deteriorates in aging ALC mice and not in their age-matched CON. We previously reported, using mice generated identically as herein, that at the onset of adult body size (age of 17 weeks) the ALC mice do not differ with respect to adiposity, fasting glucose, and glucose clearance in an IPGTT [27]. Deficits in these measures appeared by the age of 18 months; although we cannot define the age of onset, others report their emergence by 4-to-6-months of age. PAE’s impact on metabolic function is dose-dependent [28], and this study used a more moderate alcohol exposure (3 g/kg) that modestly (5%) reduces fetal weight with normal postnatal growth. Our use of a relatively low-fat diet (17% of kcals) may also have delayed its emergence, although four weeks of consumption of a high-fat diet of younger (4.5-month-old) mice did not alter adiposity, glucose tolerance, or energy expenditure in an exposure-specific manner [27]. The mechanistic basis for these changes is likely complex. Although our model does not promote fetal growth restriction, it does reduce the availability of maternal glucose for fetal use and the accompanying increase in amino acid catabolism [33] could serve as the triggering stressor in the absence of overt growth restriction. PAE can produce pancreatic, hepatic and adipocyte dysfunction in later life [12,34,35,36,37], and could be causal here as the exposure window encompassed these tissues’ induction and development. PAE also causes chronic inflammation [19,20,21] and epigenetic reprogramming [38,39], both of which are known drivers of glucose intolerance. Given the phenotype’s complexity, all are likely contributory.

As others have reported, alcohol’s impact on these metabolic outcomes is sex dependent. Although children diagnosed with FASD are more likely to be lean [1,40], perhaps because reduced growth is part of the diagnostic criteria, studies increasingly report a shift at adolescence wherein affected females but not males are more likely to be overweight or obese [12,15,16,41]. In the most comprehensive study of this question to date, involving medical chart review of 208 affected individuals and 208 case controls, adult females diagnosed with FASD were more likely to be overweight or obese (BMI ≥ 25), whereas males had higher rates of type-2 diabetes [12]. Similarly, although preclinical studies of PAE report both glucose intolerance and increased adiposity in both sexes, for the few studies that evaluate PAE littermates in head-to-head comparisons, male offspring consistently show greater insulin resistance, whereas the impact on adiposity is mixed [35,36,37]. Our study of aged littermates largely falls in line with those clinical and preclinical findings. A plausible explanation for these sex differences may lie in the distinct strategies used by male and female fetuses in response to in utero stress, wherein females reduce their growth to preserve placental efficiency and males strive to maintain their growth despite limited nutrient availability [42,43]. This may explain why males are at greater risk for adult-onset chronic disease, whereas females are better protected. In our model, the fetuses exhibit sex-dependent responses to the PAE stressor, wherein female but not male fetuses adopt an asymmetric growth strategy that correlates with litter size and expands to correlate with placental weight as the stressor intensifies [44,45].

Overall, analysis of the behavior data primarily identified sex- and age-dependent outcomes in many of the tests. Exposure effects, particularly in the fear conditioning test, were most apparent at 10 months. Although most animals were re-tested at the different ages, a small subset of animals was tested at 10 months without undergoing testing at 6-wk and their behavior outcomes were similar to the animals being re-tested. This suggests that performance differences are age-dependent and not the result of re-testing. Behavior of CON animals typically changed between 6 weeks and 10 months, whereas in ALC animals the performance was often similar at both ages. One interpretation is that moderate PAE affects cognitive behavior in adolescence and adulthood but does not precipitate a cognitive decline.

Perhaps the most important finding from this study is that the cognitive performance of individual animals correlates with their metabolic features. Some of these associations were independent of exposure; for example, the negative correlations of % body fat with T1 rotarod performance and arm entries and alternations in the Y-maze may simply reflect that increasing adiposity associates with less voluntary movement. However, other associations were unique to the ALC offspring. For example, inverse correlations were identified between % body fat and T2 rotarod performance, and % freezing to context with AUC, but only for ALC males. Similarly, AUC was inversely correlated with % time spent with a novel object in the ALC females, but positively for CON females. This could reflect that a more severe PAE outcome is more likely to impact both the metabolic and cognitive phenotype. However, in the instance of T2 rotarod, the association with AUC is parallel for ALC and CON mice but opposing when compared with % body fat, suggesting that distinct mechanisms underlie these interactions. Insight into potential mechanisms emerges from numerous studies that identify inverse correlations between cognitive performance and type-2 diabetes [46,47,48,49]. This link is thought to be mechanistic through the latter’s promotion of vascular damage, proteostasis, and a proinflammatory state, all of which reduce neuronal function and promote neuronal loss [50]. Importantly, interventions that improve metabolic function, such as caloric restriction or drugs that target insulin or mTOR signaling, delay the onset of aging-related cognitive decline [8,50]. Glucose intolerance, insulin resistance, and adiposity are the end-stage manifestations of micropathologies that emerge at earlier timepoints dating back to periconception. We propose that these associated micropathologies serve as co-morbidities that worsen cognitive outcomes in PAE and may contribute to its impairment as an individual ages. Future study will identify additional influences upon these outcome measures and additionally inform underlying mechanisms. These data emphasize the need for those with FASD to access chronic disease screening and appropriate interventions, a situation that many with FASD find challenging given their disabilities [11].

This study has several limitations. It was originally designed to evaluate behavioral outcomes in adolescent mice, and the decision to assess those behaviors, glucose metabolism, and survival was added thereafter. Thus, some measures were not continuous for every animal. The earlier deaths of some animals could have skewed the survivors to healthier animals and thus the aged results may not fully represent the starting cohort. This is being addressed in a follow-up cohort that is now underway. Nonetheless, this model has been deeply characterized with respect to fetal growth, metabolomics, gene expression, and behavior [24,25,27,33], and it thus represents an integrated preclinical platform for the exploration of the mechanisms and long-term consequences of PAE.

## 5. Conclusions

In summary, we report greater adiposity and glucose intolerance in aging ALC mice generated using a model that mirrors the diagnostic category of alcohol-related neurodevelopmental disorders. Although ALC mice have normal glucose responses and body composition at an age of 4.5 months, they show a sex-dependent worsening by age 18 months, with ALC females having greater fat mass, and ALC males having worsened glucose tolerance. These outcomes are consistent with prior work showing that PAE causes sex-specific worsening of metabolic syndrome endpoints. We did not find evidence of progressive cognitive decline in this model. However, the animal’s cognitive performance correlated with select metabolic features including body fat content and glucose clearance, suggesting that these may be mechanistically related. Overall, these data support the growing awareness that FASD should be considered a whole-body disorder.

## Data Availability

Not applicable.

## Supplementary Materials

The following supporting information can be downloaded at: https://www.mdpi.com/article/10.3390/nu14071438/s1, Figure S1: Novel Object Testing; Figure S2: % Time freezing in the fear conditioning task; Table S1: Number of animals and litters evaluated in the behavioral testing at each age; Table S2: Gestational and growth outcomes of dams and pups; Table S3: Highest % variance contributed by litter in the behavioral tests; Table S4: Identities of non-Responders in the fear conditioning tests.
