# Supplementary material for: Aging-Related Behavioral, Adiposity, and Glucose Impairments and Their Association following Prenatal Alcohol Exposure in the C57BL/6J Mouse
Source: Nutrients. 2022 Mar 30;14(7):1438. doi: 10.3390/nu14071438 (PMC9002573; doi:10.3390/nu14071438)
Supplement: Supplementary file 1 [file nutrients-14-01438-s001.zip › Supplemental Figures.pdf]

Supplemental Figure S1

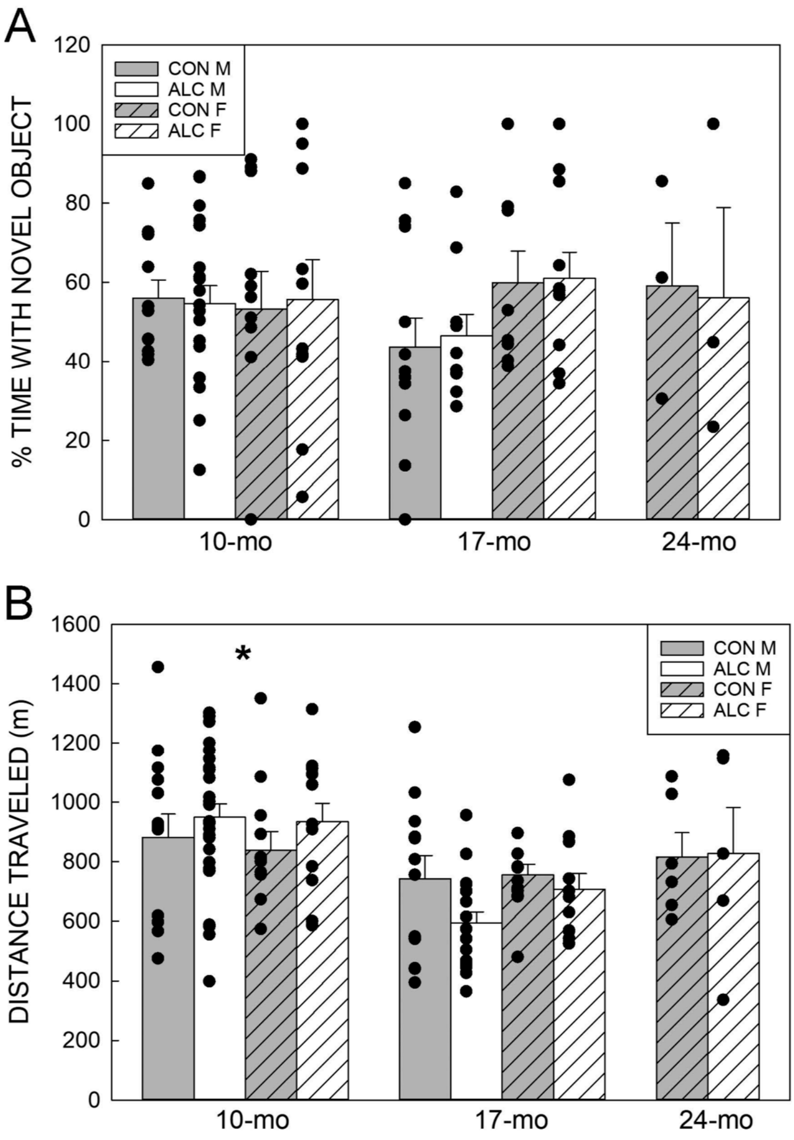

Supplemental Figure S1. Performance of ALC and CON mice in the Novel Object Recognition task. (S1A) Percentage of time spent with the novel object is not affected by exposure, sex, or age. (S1B) The distance traveled during acclimation to the arena is greater at 10-mo than at other ages tested. This task was not tested at 6-wk. Values are mean ± SEM, dots represent values for individual animals. The number of animals tested in each group is presented in Supplemental Table 1. * indicates ALC differs from CON at p≤0.05.

Supplemental Figure S2

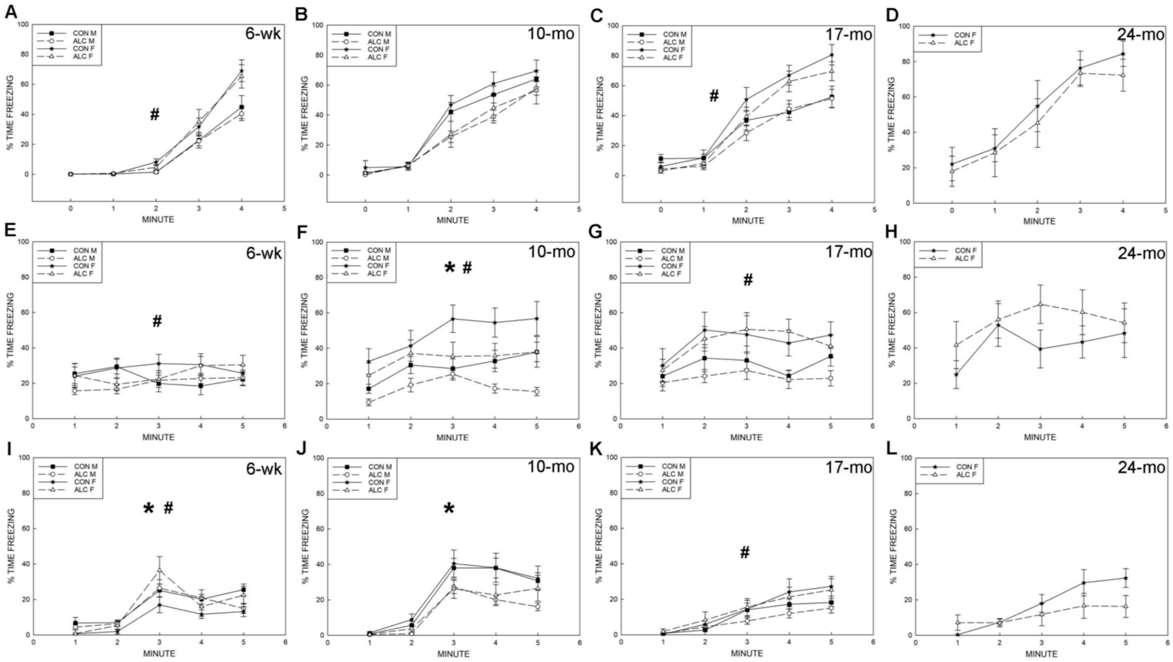

Supplemental Figure S2. Performance of ALC and CON mice in the Auditory Cued Fear Conditioning task. (A-D) Acquisition (Day 1). Shown is the percent time freezing binned for each phase of the task at ages 6-wk (S2A), 10-mo (S2B), 17-mo (S2C), and 24-mo (S2D). At all ages, all animals increase freezing with repeated exposure to the tone-shock (CSUS) pairing. (E-H) Contextual session (Day 2). Shown is the percent time freezing binned for each minute of the task at ages 6-wk (S2E), 10-mo (S2F), 17-mo (S2G), and 24-mo (S2H). (I-L) Cued session (Day 3). Shown is the percent time freezing binned for each minute of the task at ages 6-wk (S2I), 10-mo (S2J), 17-mo (S2K), and 24-mo (S2L). Most animals show increased freezing at minute 3 when the tone (CS) is played. Open symbols and dashed lines are ALC, closed symbols and solid lines are CON. Only females were tested at 24-mo. Values are mean ± SEM, dots represent values for individual animals. The number of animals tested in each group is presented in Supplemental Table 1. *, indicates ALC differs from CON at $p \leq 0.05$. #, indicates sex differences at $p < 0.05$.
