# Supplementary material for: Aging-Related Behavioral, Adiposity, and Glucose Impairments and Their Association following Prenatal Alcohol Exposure in the C57BL/6J Mouse
Source: Nutrients. 2022 Mar 30;14(7):1438. doi: 10.3390/nu14071438 (PMC9002573; doi:10.3390/nu14071438)
Supplement: Supplementary file 1 [file nutrients-14-01438-s001.zip › Supplemental Tables.pdf]

Supplemental Table S1

Number of Animals and Litters Evaluated in the Behavioral Testing at Each Age

	Rotarod	Y-maze	Novel object	Fear conditioning
CON Male 6-wk	12 (6)	12 (6)	-	10 (5)
CON Male 10-mo	14 (6)	14 (6)	11 (6)	14 (6)
CON Male 17-mo	12 (6)	12 (6)	12 (6)	10 (6)
ALC Male 6-wk	22 (6)	22 (6)	-	22 (6)
ALC Male 10-mo	24 (6)	27 (6)	20 (6)	26 (6)
ALC Male 17-mo	19 (6)	17 (5)	10 (5)	18 (6)
CON Female 6-wk	11 (7)	14 (7)	-	14 (7)
CON Female 10-mo	12 (7)	12 (7)	11 (7)	12 (7)
CON Female 17-mo	10 (7)	10 (7)	8 (7)	10 (7)
CON Female 24-mo	6 (6)	6 (6)	3 (3)	6 (6)
ALC Female 6-wk	10 (4)	10 (4)	-	11 (4)
ALC Female 10-mo	10 (4)	13 (4)	10 (4)	13 (4)
ALC Female 17-mo	11 (4)	11 (4)	11 (4)	10 (4)
ALC Female 24-mo	5 (4)	5 (4)	3 (3)	5 (4)

Number of animals (litters) for each test. CON - control. ALC – prenatal alcohol exposed.

Supplemental Table S2

Gestational and Growth Outcomes of Dams and Pups

**Dams**	**CON (7)**		**ALC (9)**	
E0.5	19.87 ± 0.317		19.51 ± 0.511	
E8.5	22.39 ± 0.553		21.52 ± 0.561	
E17.5	34.10 ± 0.780		**31.17 ± 0.895***	
Gain E0.5 – E17.5	14.23 ± 0.719		**11.66 ± 0.819***	
Gain E8.5 – E17.5	11.71 ± 0.621		**9.65 ± 0.611***	
**Pups**	**CON Males (16)**	**ALC Males (27)**	**CON Females (14)**	**ALC Females (13)**
P9	3.69 ± 0.076	**4.33 ± 0.106***	3.75 ± 0.088	4.25 ± 0.12
P15	5.80 ± 0.171	**6.85 ± 0.150***	5.90 ± 0.178	**6.94 ± 0.173***
P22	7.98 ± 0.386	**9.70 ± 0.173***	8.14 ± 0.398	**9.65 ± 0.303***
P37	18.71 ± 0.694	17.89 ± 0.477	15.72 ± 0.255	**14.76 ± 0.303***

Data shown are mean ± SEM for each group (n).
* Bold font denotes significant difference ($p<0.05$) to CON within sex / group.
Data originally presented in Mooney et al. 2021.

Supplemental Table S3

Highest % Variance Observed in Behavioral Tests

	**Highest % variance**
**Rotarod**	
T1	0
T2	11.18
**Y-maze**	
# arm entries	5.02
# alternations	0.16
% alternations	0
**Novel object**	
activity	6.14
% exploration	0.87
**Fear conditioning**	
acquisition	9.26
context	4.15
cue	6.82

Supplemental Table S4

Numbers of Non-Responder Mice in the Cued Fear Conditioning Task

<1% difference between average background freezing and average response to cue

	6wk	10mo	17mo	24mo
CON M	1			
ALC M			2	
CON F			1	1
ALC F	1	1		1

N=3 males, N=5 females

<3% difference between average background freezing and average response to cue

	6wk	10mo	17mo	24mo
CON M	1		1	
ALC M	2	3	4	
CON F	1	1	2	2
ALC F	1	2		1

N=11 males, N=10 females

<5% difference between average background freezing and average response to cue

	6wk	10mo	17mo	24mo
CON M	2 (20%)		1 (10%)	
ALC M	4 (18%)	4 (15%)	7 (39%)	
CON F	1 (7%)	1 (8%)	3 (30%)	2 (33%)
ALC F	1 (10%)	4 (30%)	1 (10%)	2 (40%)

N=18 males, N=15 females
